# Boscombe Hospital and Provident Dispensary

**Published:** 1895-09-21

**Authors:** 


### The Institutional Workshop.

### WITHIN THE HOSPITALS.

### BOSCOMBE HOSPITAL AND PROVIDENT DISPENSARY.

The well-known Victoria Hospital, which occupies so prominent a position close to the station at West Bournemouth, is apt to be thought by strangers to be the only general hospital in that rapidly increasing town; but that such is not at all the case the inhabitants of the eastern portion of the town can well testify. Rather out of the way, certainly, in Shelley Road, Boscombe, not very far from the Christchurch Road, stands the picturesque little building known as the Boscombe Hospital and Provident Dispensary, which does so much good work, and is not only much needed, but greatly appreciated, amongst the poorer population of East Bournemouth, Boscombe, Christchurch, and the surrounding district. And its claims need to be put forward with special emphasis just now, because money is very much wanted to enable necessary improvements and additions to be carried out, whereby the standard of accommodation and administration may be kept up to the pitch desired by its well-wishers.

The Boscombe Hospital has been working quietly on for many years, and with the enormously increasing population—increasing more than ever just lately in the Boscombe district—it will be easily understood that the demands upon its resources have become immensely greater, while the additional subscriptions and donations do not, unluckily, pour in with anything like proportionate rapidity. In fact, the number of beds have actually had to be reduced from the originally far too small number of twelve, to ten, the medical staff strongly representing the advisability of so doing. The reduction would have been to eight, but that by finding a bed-room outside for the house surgeon an extra ward for two beds was obtained. The best that can be made of such limited accommodation, and a more or less old building which has necessitated a good deal of cleaning, painting, and re-flooring, certainly has been made through the efforts of a committee to whom the hospital is a keen personal interest. The late hon. secretary, Mr. W. Tingle Brown, worked untiringly in its cause, and his labours are being no less energetically carried on by Mr. Evan Maberly, who has succeeded him, and whose efforts for the benefit of his institution are very evidently labours of love. It is very much to be hoped, in the interests of all concerned, that these may be attended with all success, and that funds will soon be in hand to enable the work of enlargement and rebuilding to be begun.

The present building is of the bungalow type, and the wards are very small, now containing but two beds each. This in itself is an unfortunate division, increasing the number of nurses required, and making the nursing altogether heavier than would be the case with a better distribution of beds. The out-patient and dispensary department is an important one, 1,621 new cases being treated in 1894. The number of in-patients admitted for that year was 112, of which 64 were free admissions, the rest paying according to means—the latter arrangement being one which it is to be hoped may be adhered to in any future re-constitution of the hospital's rules, rather than a reversion to the pauperising system of indiscriminating free treatment, so often unwisely held up to admiration by those responsible for hospital administration as an end to be aimed at, instead of avoided.

The accounts are kept on the uniform system; they unfortunately show a balance on the wrong side of £160 for 1894, and this Mr. Maberly is extremely anxious to clear off before embarking upon alterations. A visit to the hospital, where Mr. Maberly will gladly give details, and a talk with him and with Miss White, the matron, whose heart is no less enthusiastically in her work, would, we feel sure, speedily convert the casual visitor into a friend of a very deserving institution; and we would warmly urge all those who are interested in hospital matters not to be content with an inspection of the more imposing building which ministers to the needs of West Bournemouth, but to pursue their way to the eastern side, and after seeing the unassuming work there in progress, to do their utmost to enable the Boscombe Hospital to enlarge its sphere of usefulness with as little delay as possible.

